# Constructing and validating an Occupational Mechanical Job Exposure Index based on five Norwegian nationwide Surveys of Living Conditions on work environment

**DOI:** 10.1186/s12889-022-14460-7

**Published:** 2022-11-05

**Authors:** Åsmund Hermansen, Espen Dahl

**Affiliations:** Faculty of Social Sciences, Department of Social Work, Child Welfare and Social Policy, OsloMet – Oslo Metropolitan University, Oslo, Norway

**Keywords:** Occupational Mechanical Job Exposure Index, Job Exposure Matrix, Test of reliability, Validation, Norway

## Abstract

**Background:**

The overall aim of this study was to examine essential aspects of reliability and validity of a constructed Occupational Mechanical Job Exposure Index for use in analyses of Norwegian register data.

**Methods:**

We utilized data from the Norwegian nationwide Survey of Living Conditions on work environment in 2006, 2009, 2013, 2016 and 2019. Occupations were classified on a 4-digit level based on the Norwegian version of the International Standard Classification of Occupations (ISCO-88). We constructed a 4-digit correspondence table between the occupational codes used in the 2006 and 2009 surveys (STYRK-98) and the codes used in 2013, 2016 and 2019 (STYRK-08). The mechanical exposures were collected by Statistics Norway using telephone interviews. As for reliability, we examined the agreement between the individual- and the occupational-based mechanical exposures using Cohen’s kappa, sensitivity and specificity measures. Construct, concurrent and predictive validity pertaining to the Occupational Mechanical Job Exposure Index were analysed using both survey data and nationwide register data.

**Results:**

The analysis shows a fair-to-moderate overlap between occupational-based mechanical exposures and the individually reported exposures. Construct validity of the Occupational Mechanical Job Exposure Index, as estimated by a confirmatory factor analysis using the occupational-based mechanical exposures, showed that the 8 exposures formed one underlying factor. When assessing the concurrent value of the Occupational Mechanical Job Exposure Index to the index based on the individual reported exposures, the occupational mechanical index showed lower and reproducible associations with lower back pain for both men and women. For long-term sick leave, the occupational mechanical index showed higher and reproducible associations for both genders. As for predictive validity, the register data analysis shows that the occupational mechanical index was associated with disability and a higher number of long-term sickness benefits periods for both men and women. For men the index also predicted higher mortality.

**Conclusion:**

Our tests of reliability and validity of the Occupational Mechanical Job Exposure Index indicate that the index overall has acceptable statistical properties and will be useful in analyses of Norwegian register data where individual information on these types of exposures is missing.

**Supplementary Information:**

The online version contains supplementary material available at 10.1186/s12889-022-14460-7.

## Introduction

The Nordic countries have a longstanding tradition of using high quality register data for research purposes. The fact that these data often include the entire population and consists of long time series makes them “a goldmine” for research [1]. However, these data are not collected for research purposes and therefore often lack information that is vital for research into social inequalities in health and socialepidemiological register research in general. Varying dimensions of working conditions are usually such a “missing piece of the jigsaw puzzle” in register-based research. Knowledge about work environment is crucial in itself in a democratic society that cares about the population’s living conditions and well-being. In addition, knowledge about the pathways into and out of employment and different types of jobs is important to assess the interrelationship between work, health and wellbeing over the life course [2, 3]. One way to overcome the problem of missing information on work environment in register data is to use a job exposure matrix (JEM). Based on job titles JEM’s have been developed for a number of different and specific exposures and stressors [4–6]. Despite the great advantage of creating this kind of information for use in register data, a notorious problem with JEM is that it entails the risk of misclassification which limits its applicability. This relates to the exact definition of exposures as well as the definition of exposed/non-exposed. These possibilities and challenges related to JEM are the motivation for this undertaking.

In Norway, Hanvold et. al. [7] have constructed a JEM for mechanical and psychosocial job exposures based on the Norwegian nationwide Survey of Living Conditions on work environment in 2006 and 2009. This paper is inspired by the innovative work done by these researchers. We have, however, moved beyond Hanvold et al. in several ways. Firstly, we have added three Norwegian nationwide Survey of Living Conditions on work environment, i.e. for the years 2013, 2016 and 2019. Requiring extensive work to create a table of correspondence between the occupational codes use in the 2006 and 2009 surveys and the codes used in the 2013, 2016 and 2019 surveys. Hence, we have achieved a much larger number of observations and significantly improvement in the statistical precision of our estimates, as can be seen in the results section. Secondly, we have applied the JEM on Norwegian register data and investigate the predictive validity of the JEM using three health outcomes disability benefits, mortality, and the number of long-term sick absence periods. We have also investigated the concurrent validity of the JEM using not only lower-back pain as a health outcome, following Hanvold et. al. [7], but also including long-term sick leave. Furthermore, coming from traditions linked to social policy, health inequality research and labour market analysis, with less focus on clinical effects of single exposures, or how specific exposures are associated with specific diagnoses (e.g. [7]), we have developed a broad index for mechanical job exposures. The ultimate purpose behind the construction of the Occupational Mechanical Job Exposure Index is to create a summary measure of mechanical job exposures for use in future analyses of Norwegian register data.

### Previous research

The JEM method is quite extensively applied in a number of countries and settings. A JEM is used to assign exposures on the basis of occupational titles. Hence, a JEM is potentially convenient when information on individual occupation is available, but there is no information on job exposures or job hazards, as is the case in Norwegian register data. JEMs have been constructed, evaluated and applied in several European countries, including England [8], The Netherlands [9], Spain [10], Germany [11], France [12, 13] and also in countries overseas like e.g. the USA [14], Canada [15] and Australia [16]. Scholars in all the four Nordic countries, Denmark, Finland, Norway and Sweden, have also developed and evaluated national JEMs [7, 17–20].

Evaluating a JEM may be done by comparisons with four alternative approaches to measure exposures, i.e. self-reporting by workers themselves, expert assessments, direct measurements, and by observations of working situations [12]. In our study the basis for comparison is self-reports of mechanical exposures as obtained by representative nation-wide surveys. Work exposures may be categorized into biological, mechanical, physical, chemical and psychosocial exposures and stressors. Since our index only includes mechanical exposures, we will primarily address the literature that has examined and evaluated these kinds of exposures.

Typically, validation studies have examined reliability issues (e.g. agreement, sensitivity, specificity) and criterion related validity (e.g. concurrent validity). Some relevant systematic reviews exist. Generally, these reviews have a positive view of the usefulness of JEMs in the case that information on work environment from other sources is missing. In a systematic review of a JEM developed in the USA, the O*NET, the researchers concluded: “O*NET has the potential to allow examination of occupational risks that might have otherwise been ignored due to missing data or resource constraints on field data collection of job exposure information.” (14:898). Another systematic review claimed that: “Limited evidence also indicates that… measurement-calibrated quantitative JEMs, may be as reliable as traditional methods ([21]:1048). Similar encouraging results were also reported by Sadhra et al. [22].

Turning to countries that share many similarities with Norway, a Dutch study, Rijs et al. [23], found that use of force and work in uncomfortable positions were significantly associated with functional limitations and self-perceived health. A moderate probability of repetitive movements was related to functional limitations among former workers. The authors conclude that the results suggest that the JEM accurately classifies jobs according to physical demands.

In Denmark, Dalbøge et al. [17] validated a shoulder JEM by comparing the results from expert-rated job exposures with measured job exposures. The authors conclude that the constructed shoulder JEM was capable of presenting exposure–response relationships on measurement scales.

In Finland Solovieva et al. [5] reported that the specificity of the mechanical JEM was good, in particular among women. The degrees of agreement, measured by kappa, were fair for most exposures. For men, all JEM exposures were significantly associated with one month prevalence of low back pain. For women, this applied to four out of six JEM exposures. The researchers conclude that the JEM can be «considered as a valid instrument for exposure assessment in large-scale epidemiological studies, when more precise but more labour-intensive methods are not feasible» (5:1).

In Norway, Hanvold et al. [7] constructed and validated a JEM capturing mechanical and psychosocial work exposures. They found a general fair to moderate agreement between the JEM and individual work exposures. The concurrent validity of the JEM showed an acceptable relationship with the risk of low-back pain. The authors conclude that JEM «may be useful in large epidemiological register studies» (7: 239).

Swedish scholars constructed and assessed a summary index of physical work exposures based on three of the National Surveys of Level Livings. Reliability was shown to be acceptable in the sense that the agreement was good between individual and occupation based exposures. Also predictive validity was considered to be good as the index was related to future receipt of disability benefit [20].

In general then, assessments of reliability and criterion related validity of a variety of JEMs in a multitude of settings lead to the conclusion that they appear to be a useful, relatively inexpensive and an acceptable way to measure hazardous exposures.

Against this background, the aim of this article is to propose a Occupational Mechanical Job Exposure Index for use in Norwegian register data. This implies to assess its statistical properties in various ways, as detailed in the methods section. The idea is to use this index for different purposes in our «research program» on work, health and welfare trajectories among vulnerable groups. Hitherto, available information in Norwegian register data has been limited to occupation (job titles), social class and employment status. Our ambition is to add a reliable and validated index variable describing mechanical exposures to this list. The Occupational Mechanical Job Exposure Index is made up of eight different mechanical job exposures. The reliability of index is examined by estimating the degree of agreement between the occupation based index and an index based on individual reported exposures as well as the sensitivity and specificity of the different exposures. Construct validity of the Occupational Mechanical Job Exposure Index is assessed by a confirmatory factor analysis whereas criterion related validity (concurrent validity and predictive validity) is investigated by estimating the association between the occupational index and selected health outcomes like self-reported lower-back pain and long-term sick leave in survey data and disability and long-term sick leave in register data.

## Method

### Study population

The populations included in the analysis are described according to age, educational level and major occupational groups in Table 1 – the survey data and Table 2 – the register data. As shown in Table 1 the total population based on the survey data includes 43 977 individuals and the population based on the register data includes 1 589 535 individuals. The survey population includes all those who participated in the 2006, 2009, 2013, 2016 and 2019 Norwegian nationwide Survey of Living Conditions on work environment and had a valid occupational code. The high number of observations achieved by using respondents in five surveys is likely to increase the precision of the JEM estimates [12]. The study population based on the survey data and register data has a somewhat different age span. When defining the register data based population, we set the cut off at 55 years. The reason for this is that our analysis based on register data has a ten-year follow-up period, implying that those who were 55 will be 65 years in the last observation year. A significant share of the workforce will retire after the age of 65 years, thus no longer experiencing occupational exposures and those who do remain will most likely be a highly selected group. The survey population includes employees up till 69 years of age.

**Table 1:** Background characteristics of the study population (survey data)

	All		Men		Women	
	(*N* = 43 977)		(*N* = 23 062)		(*N* = 20 915)	
**Age (years)**	N	%	N	%	N	%
17–24	4 484	10,2	2 308	10,0	2 176	10,4
25–44	19 160	43,6	9 880	42,8	9 280	44,4
45–69	20 333	46,2	10 874	47,2	9 459	45,2
**Educational level**	N	%	N	%	N	%
Primary school	11 116	25,3	5 979	25,9	5 137	24,6
Secondary/High school	14 007	31,9	8 524	37,0	5 483	26,2
College/university 4 years	13 328	30,3	5 508	24,9	7 820	37,4
College/university > 4 years	5 366	12,2	2 969	12,9	2 397	11,5
**Major occupational groups (STYRK-98)**	N	%	N	%	N	%
Legislator, senior officials, and mangers	4 569	10,4	3 032	13,1	1 537	7,4
Professionals	7 921	18,0	4 170	18,1	3 751	17,9
Technicians and associate professionals	11 818	26,9	5 236	22,7	6 582	31,5
Clerks	2 743	6,2	1 100	4,8	1 643	7,9
Service workers, shop, and market sales workers	8 480	19,3	2 514	10,9	5 966	28,5
Skilled agricultural and fishery workers	822	1,9	670	2,9	152	0,7
Craft and related trade workers	3 911	8,9	3 665	15,9	246	1,2
Plant and machine operators and assemblers	2 552	5,8	2 235	9,7	317	1,5
Elementary occupations	1 161	2,6	440	1,9	721	3,5
**Low-back pain (previous month)**	N	%	N	%	N	%
Severely/somewhat	5 069	11,5	2 245	9,7	2 824	13,5
A little/not at all	38 908	88,5	20 817	90,3	18 091	86,5
**Long-term sick leave (previous month)**	N	%	N	%	N	%
Yes	7 046	16,0	2 946	12,8	4 100	19,6
No	36 931	84,0	20 116	87,2	16 815	80,4

**Table 2:** Background characteristics of the study population (register data)

	All		Men		Women	
	(*N* = 1 589 535)		(*N* = 819 232)		(*N* = 770 303)	
**Age (years)**	N	%	N	%	N	%
18–24	221 568	13,9	113 520	13,9	108 048	14,0
25–44	903 754	56,9	472 831	57,7	430 923	55,9
45–55	464 213	29,2	232 881	28,4	231 332	30,0
**Educational level**	N	%	N	%	N	%
Primary school	332 656	20,9	182 156	22,2	150 500	19,5
Secondary/High school	714 616	45,0	399 202	48,7	315 414	41,0
College/university 4 years	424 436	26,7	167 405	20,4	257 031	33,4
College/university > 4 years	117 827	7,4	70 469	8,6	47 358	6,2
**Major occupational groups (STYRK-98)**	N	%	N	%	N	%
Legislator, senior officials, and mangers	174 674	11,0	93 566	11,4	81 108	10,5
Professionals	188 963	12,0	101 577	12,4	87 386	11,3
Technicians and associate professionals	326 718	20,6	147 123	18,0	179 595	23,3
Clerks	125 183	7,9	50 160	6,1	75 023	9,7
Service workers, shop, and market sales workers	383 242	24,1	111 858	13,6	271 384	35,2
Skilled agricultural and fishery workers	9 810	0,6	7 176	0,9	2 634	0,3
Craft and related trade workers	170 450	10,7	161 664	19,7	8 786	1,1
Plant and machine operators and assemblers	127 104	8,0	107 531	13,1	19 573	2,5
Elementary occupations	83 391	5,24	38 577	4,7	44 814	5,8
**Disability benefits (2008–2017)**	N	%	N	%	N	%
Yes	4 878	0,31	1 939	0,24	2 939	0,38
No	1 584 657	99,69	817 293	99,76	767 364	99,62
**Mortality (2008–2017)**	N	%	N	%	N	%
Dead	18 467	1,16	11 484	1,4	6 983	0,91
Not dead	157 068	98,84	807 748	98,60	763 320	99,09
**Ten long-term sick leave periods or more (2008–2015)**	N	%	N	%	N	%
Yes	428 510	26,9	152 019	18,6	276 491	64,1
No	1 161 025	73,1	668 213	81,4	493 812	35,9

### The construction of the Job Exposure Matrix

The job exposure matrix, which forms the foundation for the Mechanical Job Exposure Index, was developed by Hanvold et. al. [7] as a gender-specific matrix with group-based exposure estimates at each intersection between the occupations (rows) and the eight mechanical exposures (columns). To achieve reliable estimates, Hanvold et. al. decided to have at least ⩾ 19 respondents with the same occupational code when constructing the JEM groups. They report that two of the authors grouped the occupations and discussed further with a third author and two experts at the Norwegian Institute of Occupational Health. In total they constructed 268 JEM-groups based on occupational codes and the answers from 18 939 respondents in the 2006 and 2009 surveys. The job exposure matrix we used when constructing the Occupational Mechanical Job Exposure Index is identical to the matrix developed by Hanvold et. al. except from the fact that we also included the 2013, 2016 and the 2019 Norwegian nationwide Survey of Living Conditions on work environment. Inclusion of these three survey populations increased the total N with 25 037 respondents and increased the mean number of respondents in each JEM group from 176 to 412. As shown in Table 3 the mean number of respondents per JEM group more than dobled in both men and woman.

**Table 3:** Number of occupational titles according to number of repsondents and number of respondents per JEM group

	All		Men		Women	
	(*N* = 333—all) (*N* = 330 – 06 & 09)		(*N* = 317) (*N* = 303 – 06 & 09)		(*N* = 281) (*N* = 268 – 06 & 09)	
**Number of occupational titles according to number of repsondents** (2006 and 2009 in brackets)	N	%	N	%	N	%
1–18	90 (148)	27 (45)	126 (164)	40 (54)	151 (190)	54 (71)
⩾ 19	243 (182)	73 (55)	191 (139)	60 (46)	130 (78)	46 (29)
Mean respondents per occupational title	132		73		74	
Min—Max respondents per occupational title	1 (1)	2224 (1075)	1 (1)	831 (343)	1 (1)	1503 (732)
**Respondents per JEM group** (2006 and 2009 in brackets)	All		Men		Women	
	(*N* = 268)		(*N* = 209)		(*N* = 195)	
Median	261 (97)		218 (78)		385 (132)	
Mean	412 (176)		276 (109)		562 (249)	
Min—Max	19 (19)	1503 (732)	19 (19)	831 (343)	19 (19)	1503 (732)

The 2006 and 2009 survey is not directly comparable to the 2013, 2016 and the 2019 in the sense that the two first surveys are based on 4-digit STYRK-98 occupational codes and the three later are based on 4-digit STYRK-08 codes. There is no official key of correspondence between the 4-digit STYRK-98 and the 4-digit STYRK-08 codes (confirmed in correspondence with Statistics Norway, section for labour market statistics), thus being able to append the five surveys we had to develop a key of correspondence. Since our register data includes the 4-digit STYRK-98 codes we choose to convert the 4-digit STYRK-08 codes in the 2013, 2016 and the 2019 survey into 4-digit STYRK-98 codes. When faced with the choice of having more than one STYRK-98 code to select, we chose to covert to the STYRK-98 code with the highest N in the 2006 and 2009 survey combined. This applied to 28 percent of the 4-digit STYRK-08 occupational codes, thus 72 percent remained unchanged.

### Mechanical job exposures

The Occupational-based Mechanical Exposure Index is based on the same eight mechanical exposures Hanvold et.al. used when constructing their gender-specific job exposure matrix (JEM). The measures used for the self-reported mechanical exposures were developed by an expert group in a Nordic project [24] and based on the scientific literature [25], the eight mechanical exposures were dichotomized into exposed and not exposed at the individual level. The questions and cut-off values used are shown in Table 4 below.

**Table 4:** Exposures, Questions and Non-exposed/Exposed. The Occupational Mechanical Job Exposure Index

**Exposures**	Questions	Not exposed/Exposed
Heavy lifting (> 20 kg)	*Do you have to lift something that weighs more than 20 kg daily, and in the case of how many times per. day?* “Yes, at least 20 times per. day”, “Yes, 5–19 times per. day”, “Yes, 1–4 times per day”, “No”	0 = Not exposed (No), 1 = Exposed (≥ 1–4 times)
Hands above shoulder height	*Do you work with your hands raised at shoulder height or higher?* – “**yes” or “no”** **If “yes”** – Can you estimate how much of the workday you do this? “almost all the time”, “about 3/4 of the time”, “about half the time”, “about 1/4 of the time”, “very little part of the time”	0 = Not exposed (no or very little of the workday), 1 = Exposed (≥ 1/4 of the workday)
Heavy physical work	*Do you work so hard that you breathe faster?* – “**yes” or “no”** **If “yes”** – Can you estimate how much of the workday you do this? “almost all the time”, “about 3/4 of the time”, “about half the time”, “about 1/4 of the time”, “very little part of the time”	0 = Not exposed (no or very little of the workday), 1 = Exposed (≥ 1/4 of the workday)
Neck flexion	*Do you work with your head forward bending?* – “**yes” or “no”** **If “yes”** – Can you estimate how much of the workday you do this? “almost all the time”, “about 3/4 of the time”, “about half the time”, “about 1/4 of the time”, “very little part of the time”	0 = Not exposed (no or very little of the workday), 1 = Exposed (≥ 1/4 of the workday)
Squatting/kneeling	*Do you have to squat or kneel when you work?* – “**yes” or “no”** **If “yes”** – Can you estimate how much of the workday you do this? “almost all the time”, “about 3/4 of the time”, “about half the time”, “about 1/4 of the time”, “very little part of the time”	0 = Not exposed (no or very little of the workday), 1 = Exposed (≥ 1/4 of the workday)
Forward bending	*Do you work in forward-leaning positions without supporting yourself with your hands or arms?* – “**yes” or “no”** **If “yes”** – Can you estimate how much of the workday you do this? “almost all the time”, “about 3/4 of the time”, “about half the time”, “about 1/4 of the time”, “very little part of the time”	0 = Not exposed (no or very little of the workday), 1 = Exposed (≥ 1/4 of the workday)
Awkward lifting	*Do you have to lift in awkward positions?* – “**yes” or “no”** **If “yes”** – Can you estimate how much of the workday you do this? “almost all the time”, “about 3/4 of the time”, “about half the time”, “about 1/4 of the time”, “very little part of the time”	0 = Not exposed (no or very little of the workday), 1 = Exposed (≥ 1/4 of the workday)
Standing/walking	*Do you work standing or walking?* – “**yes” or “no”** **If “yes”** – Can you estimate how much of the workday you do this? “almost all the time”, “about 3/4 of the time”, “about half the time”, “about 1/4 of the time”, “very little part of the time”	0 = Not exposed (≤ 1/4 of the workday), 1 = Exposed (≥ 1/2 of the workday)

All the exposure variables are constructed as the proportion of individuals within each JEM-group that are exposed to the specific exposure. Thus, we have constructed variables that, in principle, goes from 0 to 100 percent based variables that are dichotomous (exposed = 1, not exposed = 0). This means that occupational codes with a value of 0 on one of the variables implies that none with these occupational codes, belonging to the same JEM-group, has provided an answer that involves exposure. In contrast, the value 100 means that all respondents with that occupational code, belonging to the same JEM-group, have provided an answer that involves exposure. In total, we have 323 unique occupational codes that are used when the index is merged with register data.

### Assessing the reliability of the Occupational Mechanical Job Exposure Index

In order to assess the reliability of the Occupational Mechanical Job Exposure Index we calculated three different measures: Cohen`s Kappa, sensitivity and specificity. Cohen`s Kappa measures agreement between the group-based exposure estimates and the individual exposure estimates, taking into account that agreement may occur by chance. According to Cohen [26] the kappa values can be classified as poor (< 0.20), fair (0.21–0.40), moderate (0.41–0.60), good (0.61–0.80) and excellent (0.81–1) agreement. Sensitivity measures the proportion of individuals who are identified as exposed based on individual estimates, that are also identified as exposed using the group-based estimates. Specificity measures the proportion of individuals who are identified as unexposed based on individual estimates, that are also identified as unexposed using the group-based estimates. Furthermore, in order to assess the correspondence between the group-based exposures and the individual reported exposures we have also used Spearman`s Rho. Spearman`s Rho measures the monotonic relationships, whether linear or not, between two variables. In this paper we use Spearman`s Rho to investigate the correspondence, i.e. the rank order, between the exposures reported by the individual employee and the exposures linked to the individual using their occupational code.

### Assessing construct validity, concurrent validity and predictive validity of the Occupational Mechanical Job Exposure Index

In order to investigate the construct validity of the Occupational-based Mechanical Exposure Index, we performed a confirmatory factor analysis. The CFA model was fitted in Stata v16 and maximum likelihood was applied for model estimation.

To test the concurrent validity of the Occupational Mechanical Job Exposure Index individual reported low-back pain and long-term sick leave is used as outcome variables in the analysis based on the five surveys. Individual reported low-back pain is measured as a dummy-variable: “*Have you during the last month been bothered by lower back pain?*” “*Very or quite bothered*” = 1, “*a little or not at all bothered*” = 0. Individual reported sick leave is also measured as a dummy-variable: “*Have you during the last 12 months had continuous sick leave for more than 14 days?*” “Yes” = 1, “No” = 0.

Furthermore, the predictive validity of the Occupational Mechanical Job Exposure Index is tested merging the index to register data using receipt of disability benefit in the period 2008 to 2017, the number of long-term sick leave periods between 2008 and 2015 and mortality between 2008 and 2017 as outcome variables. “Disability” and “mortality” are both measured as dummy variables: “disabled during 2008 to 2017” = 1, “not disabled during 2008 to 2017” = 0 and “dead during 2008 to 2017” = 1, “not dead during 2008 to 2017” = 0. “Long-term sick leave periods” is measured as a s dummy variables: “10 or more sick leave periods exceeding 16 days between 2008 and 2015” = 1, “less than 10 sick leave periods exceeding 16 days between 2008 and 2015” = 0.

## Results

### Assessing the agreement between individual reported and occupational-based exposures

As shown in Table 5, Cohen`s Kappa is mostly in the range of fair-to-moderate. An exception is “work with neck flexion” wich is poor (0.18 in men and 0.16 in women). Using a cut-off of 20 percent for all the exposures except “standing/walking” with a cut-off of 50 percent, gave a sensitivity of > 50 percent for six out of eight exposures for both men and women. For women the same cut-offs gave a sensitivity of > 50 percent for six out of eight exposures. The specificity was ⩾ 75 percent for six out of eight exposures for men and five for women.

**Table 5:** Agreement between the Individual and Occupational Mechanical Job Exposure Index: Cohen`s Kappa, Sensitivity and Specificity measures (survey data)

		Men			Women		
Exposures	**Cut-off %**	**Kappa**	**Sensitivity (95 CI)**	**Specificity (95 CI)**	**Kappa**	**Sensitivity (95 CI)**	**Specificity (95 CI)**
Heavy lifting (> 20 kg)	20	0.37	82 (81 – 83)	66 (65 – 66)	0.32	68 (66 – 69)	77 (76 – 78)
Hands above shoulder height	20	0.39	69 (67 – 70)	81 (81 – 82)	0.25	44 (42 – 46)	87 (86 – 87)
Heavy physical work	20	0.31	76 (74 – 77)	70 (69 – 71)	0.22	52 (50 – 54)	80 (79 – 80)
Neck flexion	20	0.18	43 (42 – 45)	79 (78 – 80)	0.16	51 (49 – 52)	70 (69 – 70)
Squatting/kneeling	20	0.43	75 (74 – 77)	80 (79 – 81)	0.32	77 (76 – 79)	71 (70 – 72)
Forward bending	20	0.24	45 (43 – 47)	87 (86 – 87)	0.22	48 (46 – 50)	83 (82 – 83)
Awkward lifting	20	0.29	63 (61 – 65)	79 (79 – 80)	0.27	77 (76 – 79)	70 (69 – 70)
Standing/walking	50	0.56	76 (74 – 77)	81 (80 – 82)	0.61	86 (85 – 87)	75 (74 – 76)

Hanvold et. al. [7] used cut-off values when constructing their final Job Exposure Matrix, being as they`re goal was to investigate each exposures association with lower-back pain, our goal however is to construct a Occupational Mechanical Job Exposure Index for the use in register data analysis. Thus, we choose not to reduce the information in the exposures using cut-offs values but have instead used the exposure variables measuring the percentage within each occupational code that is exposed. The sensitivity and specificity measures provide a valuable insight into the different exposures performance in identifying exposed and non-exposed individuals. However, since our goal is to measure the overall mechanical exposures in each occupation, it seems more fruitful to consider occupations as more or less exposed based on the percentage reporting to be exposed in each occupation. Thus, we have chosen to keep the measures, measuring the percentage exposed and used these variables in the factor analysis, as is when constructing the Occupational Mechanical Job Exposure Index.

### Assessing the correspondence between the group-based exposures and the individual reported exposures

To test the correspondence between the exposures measured as percentage exposed within each occupation (group-based exposure) and the individual reported exposures we use Spearman`s Rho, the results from a rank correlation analysis is presented in Table 6.

**Table 6:** Rank correlation between exposures at the individual level and the occupational level – Spearman`s Rho (survey data)

	Men	Women
Occupational Mechanical Job Exposure Index	.642 (.000)	.626 (.000)
*Single exposures*		
Heavy lifting (> 20 kg)	.468 (.000)	.382 (.000)
Hands above shoulder height	.424 (.000)	.296 (.000)
Neck flexion	.202 (.000)	.193 (.000)
Heavy physical work	.394 (.000)	.380 (.000)
Squatting/kneeling	.465 (.000)	.403 (.000)
Forward bending	.283 (.000)	.284 (.000)
Awkward lifting	.349 (.000)	.357 (.000)
Standing/walking	.600 (.000)	.637 (.000)

The rank correlation between the Occupational Mechanical Job Exposure Index based on the individual reported exposures and the group-based exposures is 0.642 for men and 0.626 for women (see Table 6). Thus, the correlation between the index based individual reported exposures and the group-based exposures is strong for both genders. For each of the eight exposures the correlation between the individual reported exposure and the group-based exposure is weak for “neck flexion” and “forward bending”. Whereas the correlation is moderate for “heavy lifting”, “hands above head”, “Heavy physical work”, “Squatting/kneeling”, “Awkward lifting” and strong for “Standing/walking”. When comparing the sensitivity measures with the correlations it shows that those exposures with a low sensitivity, “work with neck flexion” and “forward bending” for both genders and “hands above shoulder” for women, also have a weaker correlation. Nevertheless, an overall correlation well above 0.60 for both genders demonstrates that the Occupational Mechanical Job Exposure Index, based on five Norwegian nationwide Survey of Living Conditions on work environment, is strongly correlated with the overall mechanical job exposures experienced at the individual level.

The internal consistency of the Occupational Mechanical Job Exposure Index is high as Cronbach`s alpha reaches the value of 0.93.

### Assessing the construct validity of the Occupational Mechanical Job Exposure Index

Model evaluation was based on chi-square tests for model fit and further model fit indices, including the root mean square error of approximation (RMSEA), the comparative fit index (CFI), the Tucker–Lewis index (TLI) and the standardised root mean square residual (SRMR). For model fit to be interpreted as ‘acceptable’, a RMSEA of < 0.05 was considered a close fit, while a RMSEA and a SRMR of up to 0.08 were considered acceptable. Comparing the fit of a target model to the fit of an independent or null model, the CFI has a cut-off for good fit CFI of ⩾0.90. A TLI of 0.95 indicates the model of interest improves the fit by 95% relative to the null model, and the cut-off for good fit was sat at TLI ⩾0.95. Furthermore, the correlations of residuals to improve model fit when fitting the nine one-factor models were considered [27, 28]. Potential model adjustments were based on modification indices as provided in the Stata output using the ‘estat gof, stats (all)’ command. To obtain a clearer idea of the data and potential problematic items, a one-factor model was fitted to the data. To test whether modifications, in terms of correlated within factor residuals, led to significant model improvement, modification indices were obtained using the ‘estat mindices’ command in Stata.

The results from fitting a one-factor model is shown in Table 7. The “Original” row shows the results when fitting the Occupational Mechanical Job Exposure Index with no cross-loadings and no correlated residuals. All factor loadings were high to very high (i.e. > 0.5; see column “Standardised factor loading” in Table 7).

**Table 7:** Construct validity: Confirmatory Factor Analysis of the Occupational Mechanical Job Exposure Index (one-factor model)

	**X**^**2**^	**P**	**RMSEA**	**CFI**	**TLI**	**SRMR**	**Correlated error**
Original	435.45	0.000	0.254	0.859	0.803	0.067	
**Heavy lifting (> 20 kg)** with **Heavy physical work**	19.18	0.084	0.043	0.998	0.994	0.015	.522
**Heavy lifting (> 20 kg)** with **Awkward lifting**							.392
**Hands above shoulder height** with **Squatting/kneeling**							.334
**Hands above shoulder height** with **Awkward lifting**							-.409
**Heavy physical work** with **Squatting/kneeling**							.183
**Neck flexion** with **Forward bending**							.758
**Neck flexion** with **Awkward lifting**							.229
**Neck flexion** with **Standing/walking**							.119
**Exposures**	**Standarised factor loading**^a^				**Standard error**		
**Share exposed—Heavy lifting (> 20 kg)**	.917				.010		
**Share exposed—Hands above shoulder height**	.866				.015		
**Share exposed—Heavy physical work**	.923				.009		
**Share exposed—Neck flexion**	.551				.039		
**Share exposed—Squatting/kneeling**	.834				.018		
**Share exposed—Forward bending**	.819				.019		
**Share exposed—Awkward lifting**	.950				.006		
**Share exposed—Standing/walking**	.830				.018		

^a^no cross-loadings and no correlated residuals

When fitting the one-factor model, correlated residuals were sequentially added to respective models, which improved each model fit significantly. As shown in Table 7, a model fit with ten modifications gave a satisfying model fit.

### Assessing the concurrent validity of the Individual and the Occupational Mechanical Job Exposure Index

As shown in Table 8, for both men and women, the unadjusted occupational index estimate is not significantly lower than the individual index (unadjusted and adjusted), thus the occupational index shows a reproduceable likelihood for lower-back pain for men. When adjusting for level of education and age, the reproduceable likelihood for lower-back pain is significantly lower for men, but still significant. The occupational index shows a reproduceable likelihood for long-term sick leave for both men and women, and the adjusted occupational index estimate does not significantly differ from the individual estimates. When comparing the results based on all the five surveys (2006, 2009, 2013, 2016 and 2019) with the results based on just the 2006 and 2009 surveys, there is a significantly improvement in the statistical precision of our estimates (ref. smaller confidence intervals). Overall, the results based on all the five surveys are also more conservative. Logistic regressions produce the same results (see appendix Table 1).

**Table 8:** Linear probability model using survey data only and individual reported lower-back pain and long-term sick leave as dependent variables. Results when not adjusting (model 1) and adjusting for level of education and age (model 2)

	**Men**		**Women**		**Men**		**Women**	
	Lower-back pain		Lower-back pain		Long-term sick leave		Long-term sick leave	
	Model 1	Model 2	Model 1	Model 2	Model 1	Model 2	Model 1	Model 2
	B (CI 95)	B (CI 95)	B (CI 95)	B (CI 95)	B (CI 95)	B (CI 95)	B (CI 95)	B (CI 95)
**Mechanical Job Exposure Index**								
**Individual index (2006–2009-2013–2016-2019)**	.143*** (.13—.16)	.133*** (.12—.15)	.203*** (.18—.22)	.190*** (.17—.21)	.152*** (.13—.17)	.139*** (.12—.16)	.208*** (.18.—.23)	.214*** (.19—.24)
**Individual index (2006–2009)**	157*** (.13—.18)	.147*** (.12—.17)	.222*** (.19—.26)	.208*** (.17—.24)	.168*** (.14—.20)	.160*** (.13—.19)	.245*** (.20—.28)	.240*** (.20—.28)
**Occupational index (2006–2009-2013–2016-2019)**	.115*** (.09—.14)	.060*** (.03—.09)	.214*** (.17—.25)	.159*** (.12-.20)	.204*** (.18 – .23)	.160*** (.13—.19)	.251*** (.21—.30)	.241*** (.19—.29)
**Occupational index (2006–2009)**	.123*** (.08—.16)	.069*** (.02—.11)	.209*** (.15—.27)	.153*** (.09-.22)	.209*** (.17—.25)	.176*** (.13-.22)	.272*** (.20—.34)	.242*** (.17—32)
**N (2006–2009-2013–2016-2019)**	23,062	23,062	20,915	20,915	23,062	23,062	20,915	20,915
**N (2006–2009)**	9877	9877	9063	9063	9877	9877	9063	9063

^**^: *p* ≤ 0.05, ***: *p* ≤ 0.01

### Assessing the predictive validity of the Occupational Mechanical Job Exposure Index

When investigating the association between the occupational index and disability 2008–2017, the occupational index predicts a higher likelihood for disability among both men and woman both before and after adjusting for age and level of education (Table 9).

**Table 9:** Linear probability model using disability 2008–2017, mortality 2008–2017 and ten or more long-term sick leave periods 2008 – 2015 as dependent variables. Results when not adjusting (model 1) and adjusting for level of education and age (model 2)

	**Men**		**Woman**	
	Occupational Mechanical Job Exposure Index (2006–2009-2013–2016-2019)		Occupational Mechanical Job Exposure Index (2006–2009-2013–2016-2019)	
	Model 1	Model 2	Model 1	Model 2
	B (CI 95)	B (CI 95)	B (CI 95)	B (CI 95)
**Disability 2008–2017**	.0012*** (.0005—.0018)	.0009** (.0001—.0016)	.0041*** (.0029—.0053)	.0057*** (.0044—.0069)
**Mortality 2008–2017**	.0033*** (.0017—.0049)	.0063*** (.0045—.0082)	.0015 (-.0002—.0033)	.0055*** (.0035—.0074)
**Ten or more long-term sick leave periods 2008 – 2015**	.3023*** (.2971—.3077)	.2404*** (.2344—.2464)	.4429*** (.4340—.4519)	.4075*** (.3980—.4169)
**N**	805 997	805 997	757 331	757 331

^**^: *p* ≤ 0.05, ***: *p* ≤ 0.01

The occupational index predicts higher mortality among men both before and after adjusting for age and level of education. For women the occupational index predicts higher mortality after adjusting for age and level of education.

The occupational index predicts a significantly higher probability of having ten or more long-term sick leave periods during 2008 to 2015 for both men and women, before and after adjusting for age and level of education. As shown in Table 9, the predicted likelihood is almost twice as high for women compared to men.

For all the three health outcomes, logistic regressions produce the same results (see appendix Table 2).

## Discussion and conclusion

In this paper we have examined key aspects of reliability and validity for a Occupational Mechanical Job Exposure Index. Our main findings may be summarized as follows: 1) As for reliability, the agreement between the individual and occupation based Mechanical Job Exposure Index and the sensitivity and specificity measures are mostly in the- fair- to- moderate range and hence acceptable. The same applies to the measure of correspondence (Spearman’s rho). The internal consistency of the Occupational Mechanical Job Exposure Index (measured by Chronbach’s alpha) is high. 2) A confirmatory factor analysis shows that the 8 items measuring different aspects of the Occupational Mechanical Job Exposure Index reflect one underlying dimension. The one-dimensional index thus appears to have acceptable construct validity. The analysis of survey data of concurrent validity of the occupational and the individual index suggests that it is associated with self-reported back pain and self-reported long term sick leave. The additional analysis of register data of the predictive validity of the Occupational Mechanical Job Exposure Index shows that it is related prospectively to the receipt of disability benefit, number of long-term sick leave periods and mortality. The validity of the Occupational Mechanical Job Exposure Index thus appears to be acceptable.

The ultimate purpose behind the construction of the Occupational Mechanical Job Exposure Index was to create a summary measure of mechanical job exposures for use in future analyses of Norwegian register data. Such a measure is of interest in itself as an indicator of quality of work and work hazards. To us, however, the scholarly interest is rather related to what kind of consequences it has to occupy a job with high levels of mechanical exposures. We are thinking of consequences in terms of job shifts, e.g. from heavy to lighter jobs, in terms of leaving the labour market, e.g. the “healthy worker” effect, and in terms of how incumbents’ health state becomes affected by the work environment. A particular focus is on multilevel interactions between individual circumstances and resources and work characteristics and how these interrelations unfold over the life course. Coming from traditions linked to social policy, health inequality research and social epidemiology we are less interested in clinical effects of single exposures, or how specific exposures are associated with specific diagnoses (e.g. [7]). Rather, we draw on broader perspectives developed within social epidemiology and health inequality research. These research interests are reflected in the ways we have constructed and evaluated the Occupational Mechanical Job Exposure Index. We have, for example, deliberately selected more health outcomes than Hanvold et al. [7] as these may be seen as both directly and indirectly associated with the index.

### Strengths and weaknesses

The comparisons between the occupational based exposures and the individual based exposures, and the internal consistency of the Occupational Mechanical Job Exposure Index suggest that these statistical properties altogether are satisfactory. Yet, since neither the JEM approach nor the self-report approach can be seen as a gold standard, fair to moderate agreement between the two does not imply that the JEM performs poorer than self-reports. Although the former is challenged by lack of individual variation, the latter may be subject to systematic bias du to self-reporting [29]. This means that the JEM may be less vulnerable to such bias, and thus more “objective”.

Another problem with the JEM approach is that job exposures are measured imprecisely. The rationale behind adding three more surveys to the two Hanvold et al. used, was to gain more precision in the exposure estimates. A strength of this study is the high number of observations that is achieved by merging five waves of The Survey of Living Conditions. This operation has resulted in 43 977 valid respondents. The mean number of respondents in each JEM group is 412. The largest JEM group include 1503 respondents and the smallest groups, of which there are only two, include 19 respondents. The confidence interval estimates shown in Table 8, clearly suggests that precision is higher in the 5 wave sample than in the 2 wave sample. As pointed out by Choi [30] there might be a trade-off between number of subjects in each JEM group and the precision of the estimated exposures: To collapse several occupational groups in order to obtain a higher number of observations, will to some extent result in lower precision. In our material, this is less of a problem since few occupational groups are collapsed and the remaining are rather “clean”, homogeneous groups. We feel we have gone to considerable length to reduce the imprecision problem although we have not been able to remove it completely.

As compared with the concurrent validity measured in the survey data, we consider the results of the analysis of predictive validity as particularly assuring. In general, predictive validity is considered more credible and trustworthy than concurrent validity due to the common variance problem in cross-sectional survey data [31]. The good predictive validity revealed by the analysis of the register data, supports the claim that the criterion related validity of our Occupational Mechanical Job Exposure Index is acceptable and hence that the index is valuable.

Our positive results resemble those of studies of national JEMs examined in other countries, referred to above. In general, these validation articles conclude that in cases where information from other sources such as self-reports is unavailable, the statistical properties of the JEMs are so favorable that they are considered “useful”. Our Occupational Mechanical Job Exposure Index was based on Norwegian surveys and official Norwegian job titles and, as mentioned, was specifically constructed for use on Norwegian register data. Thus, if it is to be used outside Norway, for instance for comparative purposes, it should be subject to renewed assessments of reliability and validity.

The validation literature cited above conclude that when individual information on job exposures is lacking, the JEM is a useful proxy. Our results seem to confirm this body of research. The statistical properties, i.e. several measures of reliability, as well as construct validity and criterion related validity of the Occupational Mechanical Job Exposure Index are overall acceptable. We conclude that the Occupational Mechanical Job Exposure Index is a valid measure of mechanical job exposures that can be useful and informative in register-based studies in Norway.  

## Supplementary Information

**Additional file 1:** **Appendix Table 1.** Logistic regression using survey data only and individual reported lower-back pain and long-term sick leave as dependent variables. Results when not adjusting (model 1) and adjusting for level of education and age (model 2). **Appendix table 2.** Logistic regression using disability 2008-2017 as dependent variable. Results when not adjusting (model 1) and adjusting for level of education and age (model 1). Register data.

## Data Availability

The data that support the findings of this study are available from Statistics Norway (SSB), but restrictions apply to the availability of these data, which were used under license for the current study, and so are not publicly available. Data are however available from the authors upon reasonable request and with permission of the Norwegian Data Protection Official for the Research (NSD), the Norwegian Data Protection Authority (Datatilsynet) and Statistics Norway (SSB).

## Abbreviations

STYRK-98: Standard for occupational classification used from 1998
STYRK-08: Standard for occupational classification used from 2008
JEM: Job Exposure Matrix
p: Probability
CFA: Confirmative factor analysis
RMSEA: Root mean square error of approximation
CFI: Comparative fit index
TLI: Tucker–Lewis index
SRMR: Standardised root mean square residual
