# Supplementary material for: Constructing and validating an Occupational Mechanical Job Exposure Index based on five Norwegian nationwide Surveys of Living Conditions on work environment
Source: BMC Public Health. 2022 Nov 5;22:2028. doi: 10.1186/s12889-022-14460-7 (PMC9637319; doi:10.1186/s12889-022-14460-7)
Supplement: Supplementary file 1 — Additional file 1: Appendix Table 1. Logistic regression using survey data only and individual reported lower-back pain and long-term sick leave as dependent variables. Results when not adjusting (model 1) and adjusting for level of education and age (model 2). Appendix table 2. Logistic regression using disability 2008-2017 as dependent variable. Results when not adjusting (model 1) and adjusting for level of education and age (model 1). Register data. [file 12889_2022_14460_MOESM1_ESM.docx]

## Appendix

Appendix table 1. Logistic regression using survey data only and individual reported lower-back pain and long-term sick leave as dependent variables. Results when not adjusting (model 1) and adjusting for level of education and age (model 2).

|  | Men  Lower-back pain | | Woman  Lower-back pain | | Men  Long-term sick leave | | Woman  Long-term sick leave | |
| --- | --- | --- | --- | --- | --- | --- | --- | --- |
|  | Model 1  OR  (CI 95) | Model 2  OR  (CI 95) | Model 1  OR  (CI 95) | Model 2  OR  (CI 95) | Model 1  OR  (CI 95) | Model 2  OR  (CI 95) | Model 1  OR  (CI 95) | Model 2  OR  (CI 95) |
| Mechanical Job Exposure Index |  |  |  |  |  |  |  |  |
| Individual index  (2006-2009-2013-2016-2019) | 3.98***  (3.4 – 4.6) | 3.52***  (3.0 – 4.2) | 4.55***  (3.9 – 5.4) | 4.12***  (3.5 – 4.9) | 3.31***  (2.9 – 3.8) | 2.98***  (2.5 – 3.5) | 3.34***  (2.9 – 3.9) | 3.47***  (3.0 – 4.1) |
| Individual index  (2006-2009) | 3.91***  (3.1 – 4.9) | 3.51***  (2.7 – 4.5) | 4.76***  (3.7 – 6.1) | 4.3***  (3.3 – 5.6) | 3.5***  (2.9 – 4.4) | 3.29***  (2.6 – 4.1) | 3.85***  (3.1 – 4.8) | 3.74***  (3.0 – 4.7) |
| Occupational index  (2006-2009-2013-2016-2019) | 3.46***  (2.6 – 4.5) | 1.87***  (1.4 – 2.5) | 6.29***  (4.5 – 8.8) | 3.79***  (2.7-5.4) | 5.57***  (4.4 – 7.1) | 3.69***  (2.8 – 4.8) | 4.94***  (3.7 – 6.6) | 4.47***  (3.3 – 6.0) |
| Occupational index  (2006-2009) | 3.2***  (2.2 – 4.7) | 1.88***  (1.2 – 2.9) | 5.35***  (3.3 – 8.7) | 3.30***  (2.0-5.5) | 5.28***  (3.8 – 7.4) | 3.92***  (2.7 – 5.7) | 5.13***  (3.4 – 7.8) | 4.18***  (2.7 – 6.5) |
|  |  |  |  |  |  |  |  |  |
| N  (2006-2009-2013-2016-2019) | 23062 | 23062 | 20915 | 20915 | 23062 | 23062 | 20915 | 20915 |
| N  (2006-2009) | 9877 | 9877 | 9063 | 9063 | 9877 | 9877 | 9063 | 9063 |
| **: p ≤ 0.05, ***: p ≤ 0.01 |  |  |  |  |  |  |  |  |

Appendix table 2. Logistic regression using disability 2008-2017 as dependent variable. Results when not adjusting (model 1) and adjusting for level of education and age (model 1). Register data.

|  | Men  Occupational Mechanical Job Exposure Index  (2006-2009-2013-2016-2019) | | Woman  Occupational Mechanical Job Exposure Index  (2006-2009-2013-2016-2019) | |
| --- | --- | --- | --- | --- |
|  | Model 1  OR  (CI 95) | Model 2  OR  (CI 95) | Model 1  OR  (CI 95) | Model 2  OR  (CI 95) |
| Disability 2008-2017 | 1.66***  (1.3 – 2.2) | 1.28  (.94 – 1.7) | 3.02***  (2.2 – 4.1) | 2.57***  (1.9 – 3.5) |
| Mortality 2008-2017 | 1.27***  (1.2 – 1.4) | 1.49***  (1.3 – 1.7) | 1.19  (.97 – 1.4) | 1.37***  (1.12 – 1.67) |
| Ten or more long-term sick leave periods 2008 – 2015 | 6.91***  (6.7 – 7.1) | 4.58***  (4.4 – 4.8) | 7.09***  (6.8 – 7.4) | 6.00***  (5.8 – 6.3) |
| N | 805 997 | 805 997 | 757 331 | 757 331 |
| **: p ≤ 0.05, ***: p ≤ 0.01 | | | | |
